# Supplementary material for: Epidemiology of Viral Hepatitis in the Indigenous Populations of the Arctic Zone of the Republic of Sakha (Yakutia)
Source: Microorganisms. 2024 Feb 25;12(3):464. doi: 10.3390/microorganisms12030464 (PMC10972206; doi:10.3390/microorganisms12030464)
Supplement: Supplementary file 1 [file microorganisms-12-00464-s001.zip › microorganisms-2890082-supplementary.pdf]

Supplementary Table S1. Description of commercial serological assays used in the study

Infection	Diagnostic marker	Test System (Manufacturer)	Antigens or antibodies used in the assay	Analytical sensitivity (limit of detection)	Diagnostic sensitivity	Specificity
HAV	Anti-HAV IgG	Vectohep A-IgG (Vector-Best, Novosibirsk, Russia)	HAV antigen	1.0 mIU/ml, positivity threshold 20 mIU/ml	100%	100%
HEV	Anti-HEV IgG	DS-EIA-ANTI-HEV-G (Diagnostic Systems, Nizhniy Novgorod, Russia)	2 recombinant antigens: pB166 (ORF2) and MP-II (short regions of ORF2 and ORF3)	0.25 IU/ml	100%	98.2%
	Anti-HEV IgM	DS-EIA-ANTI-HEV-M (Diagnostic Systems, Nizhniy Novgorod, Russia)	2 recombinant antigens: pB166 (ORF2) and MP-II (short regions of ORF2 and ORF3)	9 Walter Reed units/ml	98%	95.2% (Diagnostic specificity panel) 97.2% (serum samples)
HBV	HBsAg	HBsAg-EIA-BEST (Vector-Best, Novosibirsk, Russia)	Monoclonal antibodies to HBsAg	0.01 IU/ml	100%	100%
	Total Anti-HBc (IgG+IgM)	VectoHbcAg-Antibody (Vector-Best, Novosibirsk, Russia)	Recombinant HBcAg antigen	No data	100%	100%
HDV	Total Anti-HDV (IgG+IgM)	Vectohep D-antibodies (Vector-Best, Novosibirsk, Russia)	HDV recombinant antigen	Titer no less than 1:320 of the manufacturer's standard for anti-HDV antibodies	No data	100%
HCV	Total Anti-HCV (IgG+IgM)	EIA-ANTI-HCV 2.0 (Diagnostic Systems, Nizhniy Novgorod, Russia)	Peptides of core, NS3, NS4, NS5 proteins	No data	100%	100% (Diagnostic specificity panel) 99,82% (serum samples)

Note: Data presented in the table are obtained from manufacturer's inserts for respective kits

Supplementary Table S2. Sequences, pool numbers and concentrations in PCR mix of primers. Nucleotide sequences specific to HBV DNA are underlined. Nextera DNA Indexes i7 and i5 are available online: https://dnatech.genomecenter.ucdavis.edu/wp-content/uploads/2019/03/illumina-adapter-sequences-2019-1000000002694-10.pdf

Primer	Sequence	Pool	Concentration in PCR mix
HBV-F1	TCGTCGGCAGCGTCAGATGTGTATAAGAGACAGCTGGTGGCTCCAGTTCAG	1	60 nM
HBV-R1	GTCTCGTGGGCTCGGAGATGTGTATAAGAGACAGGAGGACAACAGGTTGGTGAG	1	60 nM
HBV-F3	TCGTCGGCAGCGTCAGATGTGTATAAGAGACAGGCCCGTTTGTCCTCTAATTC	1	40 nM
HBV-R3	GTCTCGTGGGCTCGGAGATGTGTATAAGAGACAGCCAGACAGTGGGGGAAAG	1	40 nM
HBV-F5	TCGTCGGCAGCGTCAGATGTGTATAAGAGACAGCACAAAACAAAAAGATGGGG	1	80 nM
HBV-R5-1	GTCTCGTGGGCTCGGAGATGTGTATAAGAGACAGGAAAGGCCTTGTAAGTTGGCGA	1	80 nM
HBV-R5-2	GTCTCGTGGGCTCGGAGATGTGTATAAGAGACAGGAAAGGCCTTATACGTTGGCGA	1	80 nM
HBV-F7	TCGTCGGCAGCGTCAGATGTGTATAAGAGACAGCTNTGCCAAGTGTTTGCTGA	1	80 nM
HBV-R7	GTCTCGTGGGCTCGGAGATGTGTATAAGAGACAGGCGMAGGATCCAGTTGGC	1	80 nM
HBV-F9	TCGTCGGCAGCGTCAGATGTGTATAAGAGACAGGGGCGCACCTCTCTTTAC	1	60 nM
HBV-R9	GTCTCGTGGGCTCGGAGATGTGTATAAGAGACAGAAGTATGCCTCAAGGTCGG	1	60 nM
HBV-F11	TCGTCGGCAGCGTCAGATGTGTATAAGAGACAGGGAGGCTGTAGGCATAAATTG	1	160 nM
HBV-R11-1	GTCTCGTGGGCTCGGAGATGTGTATAAGAGACAGCAAAAACGAGAGTAACTCCACAG	1	160 nM
HBV-R11-2	GTCTCGTGGGCTCGGAGATGTGTATAAGAGACAGGGCAAAAAAGAGAACAATTCCACAG	1	160 nM
HBV-F13-1	TCGTCGGCAGCGTCAGATGTGTATAAGAGACAGCTCTAGCYACCTGGGTGGG	1	40 nM
HBV-F13-2	TCGTCGGCAGCGTCAGATGTGTATAAGAGACAGATCTAGCYACCTGGGTGGG	1	40 nM
HBV-R13	GTCTCGTGGGCTCGGAGATGTGTATAAGAGACAGTGCGAGGCGAGGGAGTTC	1	40 nM
HBV-F15	TCGTCGGCAGCGTCAGATGTGTATAAGAGACAGCAATCGCCGCGTCGCAGA	1	240 nM
HBV-R15-1	GTCTCGTGGGCTCGGAGATGTGTATAAGAGACAGCCCTTATCCAATGGTAAATATTTNGT	1	240 nM
HBV-R15-2	GTCTCGTGGGCTCGGAGATGTGTATAAGAGACAGTTTCTCTAGGGGCAAATATTTNGT	1	240 nM
HBV-R15-3	GTCTCGTGGGCTCGGAGATGTGTATAAGAGACAGGATACCTTTGTCTAATGGTAAATATTTNGT	1	240 nM
HBV-F17-1	TCGTCGGCAGCGTCAGATGTGTATAAGAGACAGCTATTTACACACTCTATGGAAGGC	1	80 nM
HBV-F17-2	TCGTCGGCAGCGTCAGATGTGTATAAGAGACAGCATTATTTACATACTCTTTGGAAGGC	1	80 nM
HBV-F17-3	TCGTCGGCAGCGTCAGATGTGTATAAGAGACAGATTTACACACCCTATGGAAGGC	1	80 nM
HBV-R17-1	GTCTCGTGGGCTCGGAGATGTGTATAAGAGACAGTTCGGGAAAGAATCCCAGAGG	1	80 nM
HBV-R17-2	GTCTCGTGGGCTCGGAGATGTGTATAAGAGACAGCGGGAACACATCCCAGAGG	1	80 nM
HBV-F19-1	TCGTCGGCAGCGTCAGATGTGTATAAGAGACAGCGCAAATCCAGATTGGGAC	1	60 nM
HBV-F19-2	TCGTCGGCAGCGTCAGATGTGTATAAGAGACAGAGCAGTCCCGACTGGGAC	1	60 nM
HBV-R19-1	GTCTCGTGGGCTCGGAGATGTGTATAAGAGACAGATCTTGCAGAGTTTGGTGGAA	1	60 nM
HBV-R19-2	GTCTCGTGGGCTCGGAGATGTGTATAAGAGACAGATCTAGCAGAGCTTGATGAAA	1	60 nM
HBV-F2	TCGTCGGCAGCGTCAGATGTGTATAAGAGACAGCACAGAGTCTAGACTCGTGGTG	2	80 nM
HBV-R2-1	GTCTCGTGGGCTCGGAGATGTGTATAAGAGACAGGCAGGTTCTGCATGGTCC	2	80 nM
HBV-R2-2	GTCTCGTGGGCTCGGAGATGTGTATAAGAGACAGAGGTTCGGCAGGGTCC	2	80 nM
HBV-F4	TCGTCGGCAGCGTCAGATGTGTATAAGAGACAGCCATTTGTTCAGTGGTTCGTAG	2	120 nM
HBV-R4	GTCTCGTGGGCTCGGAGATGTGTATAAGAGACAGATCAATAGGCCTGTTTACAGGAAG	2	120 nM
HBV-F6	TCGTCGGCAGCGTCAGATGTGTATAAGAGACAGGAAAGTATGTCAAAGAATTGTGGG	2	120 nM
HBV-R6	GTCTCGTGGGCTCGGAGATGTGTATAAGAGACAGCAGTATGGATCGGCAGAGG	2	120 nM
HBV-F8	TCGTCGGCAGCGTCAGATGTGTATAAGAGACAGTCCTAGCCGCTTGTTTTG	2	200 nM
HBV-R8	GTCTCGTGGGCTCGGAGATGTGTATAAGAGACAGGTCCGGCAGATGAGAAGG	2	200 nM
HBV-F10	TCGTCGGCAGCGTCAGATGTGTATAAGAGACAGTCATGGAGACCACCGTGAAC	2	100 nM
HBV-R10	GTCTCGTGGGCTCGGAGATGTGTATAAGAGACAGTCACAGCTTGGAGGCTTGAA	2	100 nM
HBV-F12	TCGTCGGCAGCGTCAGATGTGTATAAGAGACAGATTGACCCGTATAAAGAATTTGG	2	160 nM
HBV-R12-1	GTCTCGTGGGCTCGGAGATGTGTATAAGAGACAGTCCTGAACTTTAGGCCCATATTAGT	2	160 nM
HBV-R12-2	GTCTCGTGGGCTCGGAGATGTGTATAAGAGACAGGCCTGATTTTTAAACCCATATTAGT	2	160 nM
HBV-F14	TCGTCGGCAGCGTCAGATGTGTATAAGAGACAGCGGAGTGTGGATTCGCAC	2	80 nM
HBV-R14	GTCTCGTGGGCTCGGAGATGTGTATAAGAGACAGCCCACCTTATGAGTCCAAGG	2	80 nM
HBV-F16-1	TCGTCGGCAGCGTCAGATGTGTATAAGAGACAGAAAAGAAGATTGCAATTGATTATGCC	2	160 nM
HBV-F16-2	TCGTCGGCAGCGTCAGATGTGTATAAGAGACAGAAAGAAGATTAAACTTAGTCATGCC	2	160 nM
HBV-R16-1	GTCTCGTGGGCTCGGAGATGTGTATAAGAGACAGAGATCTTGTTCCCAAGAATATGG	2	160 nM
HBV-R16-2	GTCTCGTGGGCTCGGAGATGTGTATAAGAGACAGTCGTGTTCCCGAGAATAAGG	2	160 nM
HBV-F18	TCGTCGGCAGCGTCAGATGTGTATAAGAGACAGTGGAACAAGATCTACAGCATGGG	2	60 nM
HBV-R18-1	GTCTCGTGGGCTCGGAGATGTGTATAAGAGACAGTATTGGTGGAGGCAGGAGG	2	60 nM
HBV-R18-2	GTCTCGTGGGCTCGGAGATGTGTATAAGAGACAGCGATTGGTAGAAGCAGGAGG	2	60 nM
HBV-F20	TCGTCGGCAGCGTCAGATGTGTATAAGAGACAGCCTCAGGCCATGCAGTGG	2	40 nM
HBV-R20	GTCTCGTGGGCTCGGAGATGTGTATAAGAGACAGAACACGAGMAGGGGTCCTAG	2	40 nM
Index 1 (i7) adapters	CAAGCAGAAGACGGCATACGAGAT[i7]GTCTCGTGGGCTCGG		
Index 2 (i5) adapters	AATGATACGGCGACCACCGAGATCTACAC[i5]TCGTCGGCAGCGTC		

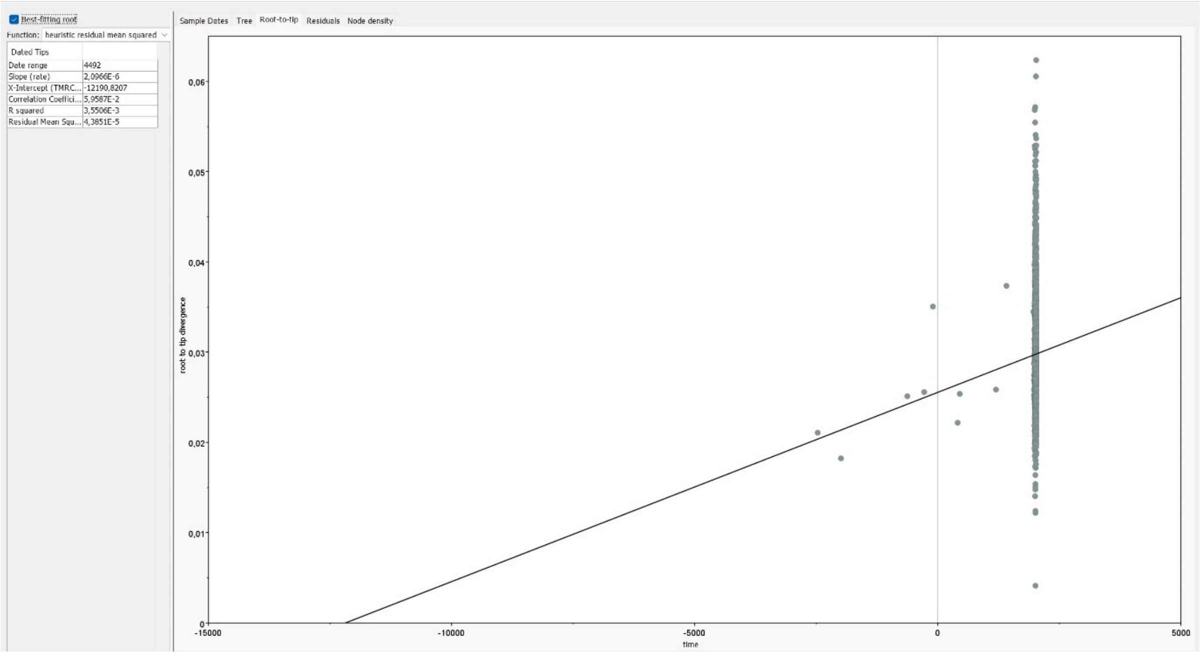

(**a**)

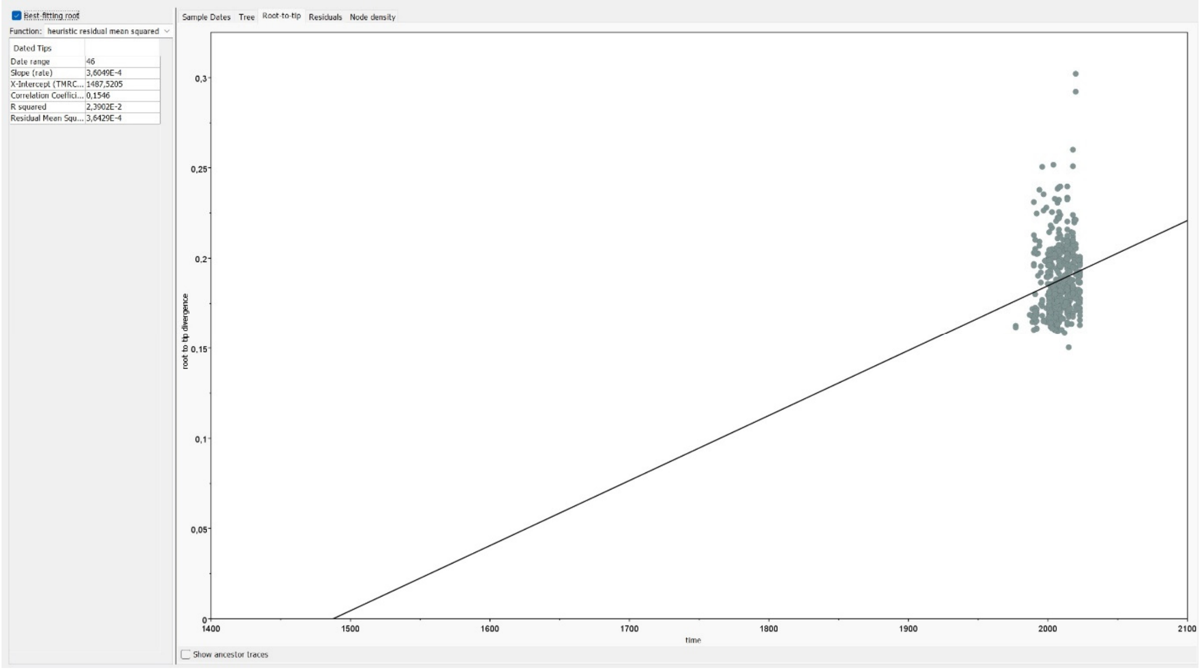

(**b**)

Supplementary Figure S1. Temporal signal linear regression graphs for HBV (a) and HCV (b) sequence datasets. The X-axis shows time in years.
